# Multiple Solutions for Nonhomogeneous Neumann Differential Inclusion Problems by the *p*(*x*)-Laplacian

**DOI:** 10.1155/2013/753262

**Published:** 2013-12-24

**Authors:** Qing-Mei Zhou

**Affiliations:** Library, Northeast Forestry University, Harbin 150040, China

## Abstract

A class of nonlinear Neumann problems driven by *p*(*x*)-Laplacian with a nonsmooth locally Lipschitz potential (hemivariational inequality) was considered. The approach used in this paper is the variational method for locally Lipschitz functions. More precisely, Weierstrass theorem and Mountain Pass theorem are used to prove the existence of at least two nontrivial solutions.

## 1. Introduction

Recently, there are several papers on the research of the Neumann-type problems involving the *p*(*x*)-Laplacian. Of the existing works in the literature, the majority deal with problems in which the potential function is smooth (i.e., *F*(*x*, ·) ∈ *C* ^1^(ℝ)). We mention the works of Mihailescu [1], Fan and Ji [2], Yao [3], Shi and Ding [4] and Cammaroto et al. [5]. Problems with a nonsmooth potential, were studied by Dai [6, 7], who for the case *N* < *p* ^−^ < +*∞* established the existence of three or infinitely many solutions for Neumann-type differential inclusion problems involving the *p*(*x*)-Laplacian, using the nonsmooth three-critical-points theorem and nonsmooth Ricceri type variational principle, respectively. Not long ago, Qian et al. [8] studied the nonhomogeneous Neumann problem with indefinite weight; that is,

(1) $$\begin{array}{c} -\mathrm{div}\left({\left|\nabla u\right|}^{p(x)-2}\nabla u\right)+V\left(x\right){\left|u\right|}^{p(x)-2}u\in \partial F\left(x,u\left(x\right)\right),\;\text{in}\;\Omega ,\\ {\left|\nabla u\right|}^{p(x)-2}\frac{\partial u}{\partial n}\in \partial G\left(x,\gamma_{0}\left(u\left(x\right)\right)\right),\;\mathrm{on}\;\partial \Omega , \end{array}$$

where *Ω* ⊂ ℝ^*N*^ is a bounded domain with smooth boundary ∂*Ω*, $\sqrt{2}p^{-}>N$, *V* ∈ *L* ^*∞*^(*Ω*) is a function possibly changing sign, *γ*_0_ : *W* ^1,*p*(*x*)^(*Ω*) → *L* ^*p*(*x*)^(∂*Ω*) is the trace operator with *γ*_0_(*u*) = *u*|_∂*Ω*_ for all *u* ∈ *W* ^1,*p*(*x*)^(*Ω*), *F*(*x*, *t*) and *G*(*x*, *t*) are locally Lipschitz functions in the *t*-variable integrand (in general it can be nonsmooth), ∂*F*(*x*, *t*) and ∂*G*(*x*, *t*) are the subdifferentials with respect to the *t*-variable in the sense of Clarke [9]. The authors prove the existence of at least one nontrivial solution of (1) using the nonsmooth Mountain Pass theorem and Weierstrass theorem.

If *V*(*x*) ≡ 1, then problem (1) becomes problem (2) as follows:

(2) $$\begin{array}{c} -\mathrm{div}\left({\left|\nabla u\right|}^{p(x)-2}\nabla u\right)+{\left|u\right|}^{p(x)-2}u\in \partial F\left(x,u\right),\;\text{in}\;\Omega ,\\ {\left|\nabla u\right|}^{p(x)-2}\frac{\partial u}{\partial n}\in \partial G\left(x,u\right),\;\mathrm{on}\;\partial \Omega . \end{array}$$

In this paper, our goal is to establish the existence of at least two nontrivial solutions for problem (2).

We emphasize that the operator −div⁡(|∇*u*|^*p*(*x*)−2^∇*u*) is said to be *p*(*x*)-Laplacian, which becomes *p*-Laplacian when *p*(*x*) ≡ *p* (a constant). The *p*(*x*)-Laplacian possesses more complicated nonlinearities than the *p*-Laplacian; for example, it is inhomogeneous and in general, it has not the first eigenvalue. The study of various mathematical problems with variable exponent growth conditions has received considerable attention in recent years. These problems are interesting in applications to modeling electrorheological fluids (see [10, 11]) and image restoration (see [12]).

This paper is divided into three sections: in the second section, we introduce some necessary knowledge on the nonsmooth analysis and basic properties of the generalized Lebesgue-space *L* ^*p*(*x*)^(*Ω*) and the generalized Lebesgue-Sobolev space *W* ^1,*p*(*x*)^(*Ω*). In the third section, we give the assumptions on the nonsmooth potentials *F*(*x*, *t*),  *G*(*x*, *t*) and prove the multiplicity results for problem (2).

## 2. Preliminary

In this section, we first review some facts on variable exponent spaces *L* ^*p*(*x*)^(*Ω*) and *W* ^1,*p*(*x*)^(*Ω*). For the details, see [13–18].

Firstly, we need to give some notations, which we will use through this paper:

(3) $$\begin{array}{c} C_{+}\left(\bar{\Omega }\right)=\left\{p\in C\left(\bar{\Omega }\right):p\left(x\right)>1\;\text{for}\;\text{any}\;x\in \bar{\Omega }\right\},\\ p^{-}=\underset{x\in \bar{\Omega }}{\min}p\left(x\right),\;\;p^{+}=\underset{x\in \bar{\Omega }}{\max}p\left(x\right)\;\text{for}\;\text{any}\;p\in C_{+}\left(\bar{\Omega }\right). \end{array}$$

Obviously, 1 < *p* ^−^ ≤ *p* ^+^ < +*∞*.

Denote by *𝒰*(*Ω*) the set of all measurable real functions defined on *Ω*. Two functions in *𝒰*(*Ω*) are considered to be one element of *𝒰*(*Ω*), when they are equal almost everywhere.

For $p\in C_{+}(\bar{\Omega })$, define

(4) $$\begin{array}{c} L^{p(x)}\left(\Omega \right)=\left\{u\in 𝒰\left(\Omega \right):\int_{\Omega }{\left|u\right|}^{p(x)}dx<+\infty \right\} \end{array}$$

with the norm

(5) $$\begin{array}{c} {\left|u\right|}_{L^{p(x)}(\Omega )}={\left|u\right|}_{p(x)}=\inf\left\{\lambda >0:\int_{\Omega }{\left|\frac{u}{\lambda }\right|}^{p(x)}dx\leq 1\right\},\\ W^{1,p(x)}\left(\Omega \right)=\left\{u\in L^{p(x)}\left(\Omega \right):\left|\nabla u\right|\in L^{p(x)}\left(\Omega \right)\right\} \end{array}$$

with the norm

(6) $$\begin{array}{c} ||u||={||u||}_{W^{1,p(x)}(\Omega )}={\left|u\right|}_{p(x)}+{\left|\nabla u\right|}_{p(x)}. \end{array}$$

Denote

(7) $$\begin{array}{c} p^{*}\left(x\right)=\{\begin{array}{cc} \frac{Np\left(x\right)}{N-p\left(x\right)}, & p\left(x\right)<N,\\ +\infty , & p\left(x\right)\geq N, \end{array}\\ p_{*}\left(x\right)=\{\begin{array}{cc} \frac{\left(N-1\right)p\left(x\right)}{N-p\left(x\right)}, & p\left(x\right)<N,\\ +\infty , & p\left(x\right)\geq N. \end{array} \end{array}$$

Let *X* be a Banach space and X* its topological dual space and we denote 〈·, ·〉 as the duality bracket for pair (X*, *X*). A function *φ* : *X* ↦ ℝ is said to be locally Lipschitz, if for every *x* ∈ *X*, we can find a neighbourhood *U* of *x* and a constant *k* > 0 (depending on *U*), such that |*φ*(*y*) − *φ*(*z*)| ≤ *k*||*y* − *z*||, for all *y*, *z* ∈ *U*.

For a locally Lipschitz function *φ* : *X* ↦ ℝ, we define

(8) $$\begin{array}{c} \varphi^{0}\left(x;h\right)=\underset{x^{\prime }\to x;\;\lambda ↓0}{\mathrm{limsup}}\frac{\varphi \left(x^{\prime }+\lambda h\right)-\varphi \left(x^{\prime }\right)}{\lambda }. \end{array}$$

It is obvious that the function *h* ↦ *φ* ^0^(*x*; *h*) is sublinear and continuous and so is the support function of a nonempty, convex, and w*-compact set ∂*φ*(*x*)⊆X*, defined by

(9) $$\begin{array}{c} \partial \varphi \left(x\right)=\left\{x^{*}\in X^{*};\langle x^{*},h\rangle \leq \varphi^{0}\left(x;h\right),\;\forall h\in X\right\}. \end{array}$$

The multifunction ∂*φ* : *X* ↦ 2^X*^ is called the generalized subdifferential of *φ*.

If *φ* is also convex, then ∂*φ*(*x*) coincides with subdifferential in the sense of convex analysis, defined by

(10) $$\begin{array}{c} \partial_{C}\varphi \left(x\right)=\left\{x^{*}\in X^{*}:\langle x^{*},h\rangle \leq \varphi \left(x+h\right)-\varphi \left(x\right)\;\forall h\in X\right\}. \end{array}$$

If *φ* ∈ *C* ^1^(*X*), then ∂*φ*(*x*) = {*φ*′(*x*)}.

A point *x* ∈ *X* is a critical point of *φ* if 0 ∈ ∂*φ*(*x*). It is easily seen that if *x* ∈ *X* is a local minimum of *φ*, then 0 ∈ ∂*φ*(*x*).

A locally Lipschitz function *φ* : *X* ↦ ℝ satisfies the nonsmooth *C*-condition at level *c* ∈ *R* (the nonsmooth *Cc*-condition for short), if for every sequence {*x*_*n*_}_*n*≥1_⊆*X*, such that *φ*(*x*_*n*_) → *c* and (1 + ||*x*_*n*_||) *m* (*x*_*n*_) → 0, as *n* → +*∞*, there is a strongly convergent subsequence, where *m* (*x*_*n*_) = inf⁡{||x*||_∗_ : x* ∈ ∂*φ*(*x*_*n*_)}. If this condition is satisfied at every level *c* ∈ ℝ, then we say that *φ* satisfies the nonsmooth *C*-condition.

**Lemma 1 (see [19])**

Consider the following.

1. The spaces *L* ^*p*(*x*)^(*Ω*) and *W* ^1,*p*(*x*)^(*Ω*) are separable and reflexive Banach spaces. Moreover, *L* ^*p*(*x*)^(*Ω*) is uniform convex.
2. If $q\in C_{+}(\bar{\Omega })$ and *q*(*x*) < p*(*x*) for any $x\in \bar{\Omega }$, then the imbedding from *W* ^1,*p*(*x*)^(*Ω*) to *L* ^*q*(*x*)^(*Ω*) is compact and continuous.
3. If *q* ∈ *C*_+_(∂*Ω*) and *q*(*x*) < *p*_∗_(*x*) for any *x* ∈ ∂*Ω*, then the imbedding from *W* ^1,*p*(*x*)^(*Ω*) to *L* ^*q*(*x*)^(∂*Ω*) is compact and continuous.

**Lemma 2 (see [15])**

The conjugate space of *L* ^*p*(*x*)^(*Ω*) is *L* ^*q*(*x*)^(*Ω*), where (1/*p*(*x*))+(1/*q*(*x*)) = 1. For any *u* ∈ *L* ^*p*(*x*)^(*Ω*) and *v* ∈ *L* ^*q*(*x*)^(*Ω*), one has

(11) $$\begin{array}{c} \int_{\Omega }\left|uv\right|dx\leq \left(\frac{1}{p^{-}}+\frac{1}{q^{-}}\right){\left|u\right|}_{L^{p(x)}(\Omega )}{\left|v\right|}_{L^{q(x)}(\Omega )}. \end{array}$$

**Lemma 3 (see [15])**

Set *I*(*u*) = ∫_*Ω*_(|∇*u*|^*p*(*x*)^ + |*u*|^*p*(*x*)^)*dx*. If *u*, *u*_*k*_ ∈ *W* ^1,*p*(*x*)^(*Ω*), then

1. for *u* ≠ 0, ||*u*|| = *λ*⇔*I*(*u*/*λ*) = 1;
2. ||*u*|| < 1( = 1; >1)⇔*I*(*u*) < 1( = 1; >1);
3. ||*u*|| > 1⇒||*u*||^*p*^−^^ ≤ *I*(*u*) ≤ ||*u*||^*p*^+^^; ||*u*|| < 1⇒||*u*||^*p*^+^^ ≤ *I*(*u*) ≤ ||*u*||^*p*^−^^;
4. lim⁡_*k*→+*∞*_||*u*_*k*_|| = 0⇔lim⁡_*k*→+*∞*_ *I*(*u*_*k*_) = 0; ||*u*_*k*_|| → +*∞*⇔*I*(*u*_*k*_)→+*∞*.

In this paper, we denote by *X* = :*W* ^1,*p*(*x*)^(*Ω*); X* = :(*W* ^1,*p*(*x*)^(*Ω*))* the dual space and by 〈·, ·〉 the dual pair. Consider the following function:

(12) $$\begin{array}{c} J\left(u\right)=\int_{\Omega }\frac{1}{p\left(x\right)}\left({\left|\nabla u\right|}^{p(x)}+{\left|u\right|}^{p(x)}\right)dx,\;u\in X. \end{array}$$

We know that (see [20]) *J* ∈ *C* ^1^(*X*, ℝ) and *p*(*x*)-Laplacian operator −Δ_*p*(*x*)_ *u* = −div⁡(|∇*u*|^*p*(*x*)−2^∇*u*) is the derivative operator of *J* in the weak sense. We denote *ℒ* = *J*′ : *X* → X*; then 〈*ℒ*(*u*), *v*〉 = ∫_*Ω*_(|∇*u*|^*p*(*x*)−2^∇*u* · ∇*v* + |*u*|^*p*(*x*)−2^ *uv*)*dx*, for all *u*, *v* ∈ *X*.

**Lemma (see [19])**

Set *X* = *W* ^1,*p*(*x*)^(*Ω*), *ℒ* is as above, then

1. *ℒ* : *X* → X* is a continuous, bounded, and strictly monotone operator;
2. *ℒ* is a mapping of type (*S*_+_); that is, if *u*_*n*_⇀*u* weakly in *X* and limsup⁡_*n*→*∞*_〈*ℒ*(*u*_*n*_), *u*_*n*_ − *u*〉≤0, implies that *u*_*n*_ → *u* in *X*;
3. *ℒ* : *X* → X* is a homeomorphism.

The following theorem, which is used as a theoretical basis in this paper, is a nonsmooth version of the well-known Mountain Pass theorem (see Chang [19] or Kourogenis and Papageorgiou [21]).

**Theorem**

Let *φ* : *X* → ℝ be locally Lipschitz function and *x*_0_, *x*_1_ ∈ *X*. If there exists a bounded open neighbourhood *U* of *x*_0_, such that *x*_1_ ∈ *X*∖*U*, max⁡{*φ*(*x*_0_), *φ*(*x*_1_)} < inf⁡_∂*U*_ *φ* and *φ* satisfies the nonsmooth *C*-condition at level *c*, where *c* = inf⁡_*γ*∈*𝒯*_max⁡_*t*∈[0,1]_ *φ*(*γ*(*t*)),  *𝒯* = {*γ* ∈ *C*([0,1]; *X*):*γ*(0) = *x*_0_, *γ*(1) = *x*_1_}, then *c* is a critical value of *φ* and *c* ≥ inf⁡_∂*U*_ *φ*.

## 3. The Main Result and Proof of the Theorem

In this section, we will discuss the existence of weak solution of (2).

Our hypotheses on nonsmooth potential *F*(*x*, *t*) and *G*(*x*, *t*) are given as follows.

 
*H*(*F*) : *F* : *Ω* × ℝ → ℝ is a function such that *F*(*x*, 0) = 0 almost everywhere on *Ω* and satisfies the following facts:
 (1) for all *t* ∈ ℝ, *x* ↦ *F*(*x*, *t*) is measurable;
 (2) for almost all *x* ∈ *Ω*, *t* ↦ *F*(*x*, *t*) is locally Lipschitz.
 
*H*(*G*) : *G* : ∂*Ω* × ℝ → ℝ is a function such that *G*(*x*, 0) = 0 almost everywhere on ∂*Ω* and satisfies the following facts:
 (1) for all *t* ∈ ℝ, *x* ↦ *G*(*x*, *t*) is measurable;
 (2) for almost all *x* ∈ ∂*Ω*, *t* ↦ *G*(*x*, *t*) is locally Lipschitz.

We consider the energy function *φ* : *X* → ℝ for problem (2), defined by

(13) φ(u)=∫Ω1p(x)[|∇u|p(x)+|u|p(x)]dx −∫ΩF(x,u)dx−∫∂ΩG(x,u)dσ, u∈X,

where *dσ* is the surface measure on ∂*Ω*.

**Lemma**

Suppose that *H*(*F*), *H*(*G*), and the following conditions hold:

(*f*_1_) there exist *c*_1_ > 0, $\alpha \in C(\bar{\Omega })$ with 1 < *α*(*x*) ≤ *α* ^+^ < *p* ^−^ such that
  (14) $$\begin{array}{c} \begin{array}{c} \left|w\right|\leq c_{1}{\left|t\right|}^{\alpha (x)-1} \end{array} \end{array}$$
 for almost all *x* ∈ *Ω*, all *t* ∈ ℝ and *w* ∈ ∂*F*(*x*, *t*);
(*g*_1_) there exist *c*_2_ > 0, *β* ∈ *C*(∂*Ω*) with 1 < *β*(*x*) ≤ *β* ^+^ < *p* ^−^ < *p*_∗_(*x*) such that
  (15) $$\begin{array}{c} \begin{array}{c} \left|w\right|\leq c_{2}{\left|t\right|}^{\beta (x)-1} \end{array} \end{array}$$
 for almost all *x* ∈ ∂*Ω*, all *t* ∈ ℝ and *w* ∈ ∂*G*(*x*, *t*).

Then, *φ* is locally Lipschitz in *X*.

**Proof**

By *J* ∈ *C* ^1^(*X*, ℝ), we have $J(u_{1})-J(u_{2})=J^{\prime }(\bar{u})\cdot (u_{1}-u_{2})$, where $\bar{u}=tu_{1}+(1-t)u_{2}$, *t* ∈ (0,1).

Let *B*_*r*_ = {*x* ∈ *X* : ||*u*−*u*_0_||_*X*_ ≤ *r*}.

Note that *B*_*r*_ is *w*-compact. Then, we obtain that there exists a positive constant *M*, such that ${||J^{\prime }(\bar{u})||}_{X^{*}}\leq M$ for sufficiently small *r*.

Therefore, for any *u*_1_, *u*_2_ ∈ *B*_*r*_, we have

(16) |J(u1)−J(u2)|=|J(u¯)·(u1−u2)|≤||J′(u¯)||X∗||u1−u2||X≤M||u1−u2||X.

On the other hand, by (*f*_1_) and Lebourg mean value theorem, we have

(17) $$\begin{array}{c} \begin{array}{c} \left|F\left(x,u_{1}\right)-F\left(x,u_{2}\right)\right|\leq c_{1}{\left|\bar{u}\right|}^{\alpha (x)-1}\left|u_{1}-u_{2}\right|. \end{array} \end{array}$$

Hence,

(18) |∫ΩF(x,u1)dx−∫ΩF(x,u2)dx|  ≤c1∫Ω|u¯|α(x)−1|u1−u2|dx  ≤c2||u¯|α(x)−1|Lα′(x)(Ω)|u1−u2|Lα(x)(Ω),

where (1/*α*′(*x*))+(1/*α*(*x*)) = 1.

Obviously, it is verified that

(19) ∫Ω(|u¯|α(x)−1)α′(x)dx=∫Ω|u¯|α(x)dx≤{|u¯|α(x)α+≤c||u¯||α+,|u¯|α(x)>1,|u¯|α(x)α−≤c||u¯||α−,|u¯|α(x)<1

is bounded, since $\bar{u}\in B_{r}$.

So,

(20) |∫ΩF(x,u1)dx−∫ΩF(x,u2)dx|  ≤c3|u1−u2|α(x)≤c||u1−u2||,

since *W* ^1,*p*(*x*)^(*Ω*)↪*L* ^*α*(*x*)^(*Ω*) is a compact imbedding.

As above, there is a positive constant *c*_4_, such that

(21) |∫∂ΩG(x,u1)dσ−∫∂ΩG(x,u2)dσ|  ≤c4|u1−u2|Lβ(x)(∂Ω)≤c||u1−u2||,

since *W* ^1,*p*(*x*)^(*Ω*)↪*L* ^*β*(*x*)^(∂*Ω*) is a compact imbedding.

Therefore, *φ* is locally Lipschitz.

**Remark**

If assumptions (*f*_1_) and (*g*_1_) in Lemma 6 are replaced, respectively, by the following:

$\left({\bar{f}}_{1}\right)$ there exist $c_{1},{\bar{c}}_{1}>0,\;\;\alpha \in C(\bar{\Omega })$ with 1 < *α*(*x*) ≤ *α* ^+^ < *p* ^−^ < p*(*x*) such that
  (22) $$\begin{array}{c} \begin{array}{c} \left|w\right|\leq c_{1}{\left|t\right|}^{\alpha (x)-1}+{\bar{c}}_{1} \end{array} \end{array}$$
 for almost all *x* ∈ *Ω*, all *t* ∈ ℝ and *w* ∈ ∂*F*(*x*, *t*);
$\left({\bar{g}}_{1}\right)$ there exist $c_{2},{\bar{c}}_{2}>0$, *β* ∈ *C*(∂*Ω*) with 1 < *β*(*x*) ≤ *β* ^+^ < *p* ^−^ < *p*_∗_(*x*), such that
  (23) $$\begin{array}{c} \begin{array}{c} \left|w\right|\leq c_{2}{\left|t\right|}^{\beta (x)-1}+{\bar{c}}_{2} \end{array} \end{array}$$
 for almost all *x* ∈ ∂*Ω*, all *t* ∈ ℝ and *w* ∈ ∂*G*(*x*, *t*), then the result of Lemma 6 is also correct.

**Theorem**

Let *H*(*F*), *H*(*G*), (*f*_1_), (*g*_1_), and the following conditions (*f*_2_)-(*f*_3_), (*g*_2_) hold:

(*f*_2_) there exist *c*_5_ > 0, *x*_0_ ∈ *Ω* and 0 < *r* < 1, such that
  (24) $$\begin{array}{c} w\geq c_{5}{\left|t-\delta \right|}^{\beta_{0}-1},\;\forall x\in B_{2r}\left(x_{0}\right),\;\;\delta <t\leq 1, \end{array}$$
 where 1 < *β*_0_ < *p* ^−^, *w* ∈ ∂*F*(*x*, *t*);
(*f*_3_) *wt* ≤ 0, *for all x* ∈ *Ω*, *w* ∈ ∂*F*(*x*, *t*) and 0 < |*t* | ≤*δ*;
(*g*_2_) *vt* ≤ 0, *for all x* ∈ ∂*Ω*, *v* ∈ ∂*G*(*x*, *t*) and 0 < |*t* | ≤*δ*,
 where
  (25) 0<δ<min⁡{12[c5β0rp+p−α−2N+β0[α−(rp++2p+)+2c1p−rp+]]1/(p−−β0),12}.

Then, the problem (2) has at least two nontrivial solutions.

**Proof**

The proof is divided into four steps as follows.

*Step  1.* We will show that *φ* is coercive in this step.

Firstly, for almost all *x* ∈ *Ω*, by *H*(*F*) (2), *t* ↦ *F*(*x*, *t*) is differentiable almost everywhere on ℝ and we have

(26) $$\begin{array}{c} \begin{array}{c} \frac{d}{dt}F\left(x,t\right)\in \partial F\left(x,t\right). \end{array} \end{array}$$

Moreover, from (*f*_1_), we have

(27) $$\begin{array}{c} F\left(x,t\right)=F\left(x,0\right)+\int_{0}^{t}\frac{d}{ds}F\left(x,s\right)ds\leq \frac{c_{1}}{\alpha \left(x\right)}{\left|t\right|}^{\alpha (x)}, \end{array}$$

for almost all *x* ∈ *Ω* and *t* ∈ ℝ.

Note that 1 < *α*(*x*) ≤ *α* ^+^ < *p* ^−^ < p*(*x*) and 1 < *β*(*x*) ≤ *β* ^+^ < *p* ^−^ < *p*_∗_(*x*); then by Lemma 1, we have *W* ^1,*p*(*x*)^(*Ω*)↪*L* ^*α*(*x*)^(*Ω*) and *W* ^1,*p*(*x*)^(*Ω*)↪*L* ^*β*(*x*)^(∂*Ω*) (compact imbedding).

Furthermore, there exist $c_{6},{\bar{c}}_{6}$ such that

(28) $$\begin{array}{c} \begin{array}{c} {\left|u\right|}_{L^{\alpha (x)}(\Omega )}\leq c_{6}||u||,\;\;{\left|u\right|}_{L^{\beta (x)}(\partial \Omega )}\leq {\bar{c}}_{6}||u||. \end{array} \end{array}$$

So, for any |*u*|_*L*^*α*(*x*)^(*Ω*)_ > 1, |*u*|_*L*^*β*(*x*)^(∂*Ω*)_ > 1 and ||*u*|| > 1, we have

(29) $$\begin{array}{c} \int_{\Omega }{\left|u\right|}^{\alpha (x)}dx\leq {\left|u\right|}_{L^{\alpha (x)}\left(\Omega \right)}^{\alpha^{+}}\leq c_{6}^{\alpha^{+}}{||u||}^{\alpha^{+}},\\ \int_{\partial \Omega }{\left|u\right|}^{\beta (x)}dx\leq {\left|u\right|}_{L^{\beta (x)}\left(\partial \Omega \right)}^{\beta^{+}}\leq {\bar{c}}_{6}^{\beta^{+}}{||u||}^{\beta^{+}}. \end{array}$$

Hence,

(30) φ(u)=∫Ω1p(x)(|∇u|p(x)+|u|p(x))dx −∫ΩF(x,u)dx−∫∂ΩG(x,u)dσ≥1p+||u||p−−c1α−c6α+||u||α+ −c2β−c¯6β+||u||β+→∞, as  ||u||→∞.

*Step  2.* We will show that the *φ* is weakly lower semicontinuous.

Let *u*_*n*_⇀*u* weakly in *W* ^1,*p*(*x*)^(*Ω*); by Lemma 1, we obtain the following results:

(31) $$\begin{array}{c} W^{1,p(x)}\left(\Omega \right)↪L^{p(x)}\left(\Omega \right),\\ W^{1,p(x)}\left(\Omega \right)↪L^{p(x)}\left(\partial \Omega \right);\\ u_{n}\to u\;\text{in}\;L^{p(x)}\left(\Omega \right),\\ u_{n}\to u\;\text{in}\;L^{p(x)}\left(\partial \Omega \right);\\ u_{n}\left(x\right)\to u\left(x\right)\;\text{a}.\text{a}.\;x\in \Omega ,\\ u_{n}\left(y\right)\to u\left(y\right)\;\text{a}.\text{a}.\;y\in \partial \Omega ;\\ F\left(x,u_{n}\left(x\right)\right)\to F\left(x,u\left(x\right)\right)\;\text{a}.\text{a}.\;x\in \Omega ,\\ G\left(x,u_{n}\left(y\right)\right)\to G\left(x,u\left(y\right)\right)\;\text{a}.\text{a}.\;y\in \partial \Omega . \end{array}$$

By Fatou's lemma, we have

(32) $$\begin{array}{c} \underset{n\to \infty }{\mathrm{limsup}}\int_{\Omega }F\left(x,u_{n}\left(x\right)\right)dx\leq \int_{\Omega }F\left(x,u\left(x\right)\right)dx,\\ \underset{n\to \infty }{\mathrm{limsup}}\int_{\partial \Omega }G\left(x,u_{n}\left(x\right)\right)d\sigma \leq \int_{\partial \Omega }G\left(x,u\left(x\right)\right)d\sigma . \end{array}$$

Thus,

(33) liminf⁡n→∞φ(un)=liminf⁡n→∞J(un)−limsup⁡n→∞∫ΩF(x,un)dx −limsup⁡n→∞∫∂ΩG(x,un)dσ≥J(u) −∫ΩF(x,u)dx−∫∂ΩG(x,u)dσ=φ(u).

Hence, by Weierstrass theorem, we deduce that there exists a global minimizer *u*_0_ ∈ *W* ^1,*p*(*x*)^(*Ω*) such that

(34) $$\begin{array}{c} \begin{array}{c} \varphi \left(u_{0}\right)=\underset{u\in W^{1,p(x)}\left(\Omega \right)}{\min}\varphi \left(u\right). \end{array} \end{array}$$

*Step  3.* In this step, we prove that *φ*(*u*_0_) < 0.

By (*f*_2_), we have

(35) $$\begin{array}{c} F\left(x,t\right)\geq \frac{c_{5}}{\beta_{0}}{\left|t-\delta \right|}^{\beta_{0}}-\frac{c_{1}}{\alpha^{-}}\delta^{\alpha (x)},\;\forall x\in B_{2r}\left(x_{0}\right),\;\;\delta <t\leq 1. \end{array}$$

Suppose that *x*_0_ ∈ *Ω* and *B*_2*r*_(*x*_0_)⊆*Ω* with 2*r* < 1. Let *η* ∈ *C*_0_^*∞*^(*B*_2*r*_(*x*_0_)) such that *η* = 1, *x* ∈ *B*_*r*_(*x*_0_); 0 ≤ *η*(*x*) ≤ 1 and |∇*η* | ≤2/*r*. Denote *s* = 2*δ*; then

(36) φ(sη)=∫Ω1p(x)(|∇η|p(x)sp(x)+|η|p(x)sp(x))dx −∫ΩF(x,sη)dx−∫∂ΩG(x,sη)dσ=∫B2r(x0)1p(x)(|∇η|p(x)sp(x)+|η|p(x)sp(x))dx −∫B2r(x0)F(x,sη)dx=∫B2r(x0)1p(x)(|∇η|p(x)sp(x)+|η|p(x)sp(x))dx −[∫B2r(x0)∩{x ∣ 0<η≤(1/2)}F(x,sη)dx   +∫B2r(x0)∩{x ∣ (1/2)<η≤1}F(x,sη)dx]≤1p−[(2r)p++1]∫B2r(x0)sp(x)dx +c1α−∫B2r(x0)∩{x ∣ 0<η≤(1/2)}|s|α(x)|η|α(x)dx +c1α−∫B2r(x0)∩{x ∣ (1/2)<η(x)≤1}δα(x)dx −∫B2r(x0)∩{x ∣ (1/2)<η≤1}c5β0sβ0(η−12)β0dx≤{1p−[(2r)p++1]sp−+c1α−sα−+c1α−δα−} ×meas⁡(B2r(x0))−∫Br(x0)c5β0sβ0(12)β0dx≤{1p−[(2r)p++1]sα−+c1α−sα−+c1α−δα−} ×meas⁡(B2r(x0))−c5β0(12)β0meas⁡(Br(x0))=meas⁡(Br(x0)) ×{[1p−[(2r)p++1]sα−+c1α−sα−+c1α−δα−]2N   −c5β0(12)β0sβ0}=meas⁡(Br(x0)) ×{[1p−[(2r)p++1]sα−+c1α−sα−+c1α−sα−2α−]2N   −c5β0(12)β0sβ0}<sβ0meas⁡(Br(x0)) ×{[1p−[(2r)p++1]+c1α−+c1α−]sα−−β02N   −c5β0(12)β0}<0.

*Step  4.* We will show that there exists another nontrivial weak solution of problem (2).

Let $\rho \in C(\bar{\Omega })$ and *p* ^+^ < *ρ* ^−^ ≤ *ρ* ^+^ < *p*_∗_(*x*).

For *u* ∈ *X* with ||*u*|| < 1, by Lemma 1, we have

(37) $$\begin{array}{c} \int_{\Omega }{\left|u\right|}^{\rho (x)}dx\leq c_{7}{||u||}^{\rho^{-}},\;\;\int_{\partial \Omega }{\left|u\right|}^{\rho (x)}dx\leq {\bar{c}}_{7}{||u||}^{\rho^{-}}, \end{array}$$

where *c*_7_ and ${\bar{c}}_{7}$ are two positive constants.

By (*f*_3_), we have

(38) $$\begin{array}{c} \begin{array}{c} 0\in \partial F\left(x,0\right),\;\forall x\in \Omega . \end{array} \end{array}$$

From Lebourg's mean value theorem and (*f*_3_), we obtain

(39) F(x,t)−F(x,0)∈〈∂F(x,λt),t〉  =1λ〈∂F(x,λt),λt〉≤0,

where *λ* ∈ (0,1), 0 < |*t* | ≤*δ*.

Thus,

(40) $$\begin{array}{c} \begin{array}{c} F\left(x,t\right)\leq F\left(x,0\right)=0,\;\forall x\in \Omega ,\;\;0<\left|t\right|\leq \delta . \end{array} \end{array}$$

Set

(41) ||u0||=r0,d=min⁡{r02,12c7,12c¯7,      (α−β−2p+[c1c7β−+c2c¯7α−]δ(α−−ρ+))1/(ρ−−p−)}.

Divide *Ω* into two parts: *Ω*_1_ = {*x* ∈ *Ω* : 0 < |*u*(*x*)|≤*δ*} and *Ω*_2_ = {*x* ∈ *Ω* : |*u*(*x*)|≥*δ*}.

For any *u* ∈ *X* such that ||*u*|| = *d*, we have

(42) ∫ΩF(x,u)dx=∫Ω1F(x,u)dx+∫Ω2F(x,u)dx≤∫Ω2F(x,u)dx≤c1α−∫Ω2|u|α(x)dx=c1α−∫Ω2|u|ρ(x)|u|α(x)−ρ(x)dx≤c1α−δ(α−−ρ+)∫Ω2|u|ρ(x)dx≤c1α−δ(α−−ρ+)∫Ω|u|ρ(x)dx.

As above, we have

(43) $$\begin{array}{c} \int_{\partial \Omega }G\left(x,u\right)d\sigma \leq \frac{c_{2}}{\beta^{-}}\delta^{\left(\beta^{-}-\rho^{+}\right)}\int_{\partial \Omega }{\left|u\right|}^{\rho (x)}dx. \end{array}$$

Hence,

(44) φ(u)=∫Ω1p(x)(|∇u|p(x)+|u|p(x))dx −∫ΩF(x,u)dx−∫∂ΩG(x,u)dσ≥1p+||u||p−−c1α−c7δ(α−−ρ+)||u||ρ− −c2β−c¯7δ(α−−ρ+)||u||ρ−≥1p+||u||p−(1−[c1α−c7+c2β−c¯7]δ(α−−ρ+)p+||u||ρ−−p−)≥12p+dp−>0.

Note that *φ* is coercive; hence, it satisfies the nonsmooth *C*-condition. So by the nonsmooth Mountain Pass theorem (consequence of Theorem 5), there exists a *u*_1_ ∈ *W* ^1,*p*(*x*)^(*Ω*) such that

(45) $$\begin{array}{c} \begin{array}{c} \varphi \left(u_{1}\right)=c>0,\;\;m\left(u_{1}\right)=0, \end{array} \end{array}$$

which means that *u*_1_ is another nontrivial critical point of *φ*.

Using the similar and simpler arguments, we can prove the following theorems.

**Theorem**

Let *H*(*F*), *H*(*G*), (*f*_1_), (*f*_2_), (*g*_1_), and the following conditions (*f*_3_′), (*g*_2_′) hold:

(*f*_3_′) *F*(*x*, *t*) = 0, *for all x* ∈ *Ω*, 0 < |*t* | ≤*δ*;
(*g*_2_′) *G*(*x*, *t*) = 0, *for  all* *x* ∈ ∂*Ω*, 0 < |*t* | ≤*δ*,
 where
  (46) $$\begin{array}{c} 0<\delta <\;\min\left\{\frac{1}{2}{\left[\frac{c_{5}r^{p^{+}}p^{-}}{\beta_{0}2^{N+\beta_{0}}\left(r^{p^{+}}+2^{p^{+}}\right)}\right]}^{1/\left(p^{-}-\beta_{0}\right)},\frac{1}{2}\right\}. \end{array}$$

Then, the problem (2) has at least two nontrivial solutions.

**Proof**

The steps are similar to those of Theorem 8. In fact, we only need to modify Step  3 as follows: (3′) show that the *φ*(*u*_0_) < 0 under the assumptions of Theorem 9. Then, from Steps  1, 2, 3′, and 4 above, the problem (2) has at least two nontrivial solutions.

Step 3′. By (*f*_2_), we have

(47) $$\begin{array}{c} \begin{array}{c} F\left(x,t\right)\geq \frac{c_{5}}{\beta_{0}}{\left|t-\delta \right|}^{\beta_{0}},\;\forall x\in B_{2r}\left(x_{0}\right),\;\;\delta <t\leq 1. \end{array} \end{array}$$

Suppose that *x*_0_ ∈ *Ω* and *B*_2*r*_(*x*_0_)⊆*Ω* with 2*r* < 1.

Let *η* ∈ *C*_0_^*∞*^(*B*_2*r*_(*x*_0_)) such that *η* = 1, *x* ∈ *B*_*r*_(*x*_0_); 0 ≤ *η*(*x*) ≤ 1 and |∇*η* | ≤(2/*r*). Denote *s* = 2*δ*; then

(48) φ(sη)=∫Ω1p(x)(|∇η|p(x)sp(x)+|η|p(x)sp(x))dx −∫ΩF(x,sη)dx−∫∂ΩG(x,sη)dσ=∫B2r(x0)1p(x)(|∇η|p(x)sp(x)+|η|p(x)sp(x))dx −∫B2r(x0)F(x,sη)dx=∫B2r(x0)1p(x)(|∇η|p(x)sp(x)+|η|p(x)sp(x))dx −[∫B2r(x0)∩{x ∣ 0<η≤(1/2)}F(x,sη)dx  +∫B2r(x0)∩{x ∣ (1/2)<η≤1}F(x,sη)dx]≤1p−[(2r)p++1]∫B2r(x0)sp(x)dx −∫B2r(x0)∩{x ∣ (1/2)<η≤1}c5β0sβ0(η−12)β0dx≤1p−[(2r)p++1]sp−meas⁡(B2r(x0)) −∫Br(x0)c5β0sβ0(12)β0dx=1p−[(2r)p++1]sp−meas⁡(B2r(x0)) −c5β0(12)β0meas⁡(Br(x0))=meas⁡(Br(x0)) ×{[1p−(2r)p++1]sp−2N−c5β0(12)β0sβ0}=sβ0meas⁡(Br(x0)) ×{1p−[(2r)p++1]sp−−β02N−c5β0(12)β0}<0.

This is the end.

**Corollary**

Let *H*(*F*), *H*(*G*), (*f*_2_),  $\left({\bar{f}}_{1}\right)$, and $\left({\bar{g}}_{1}\right)$ hold; then the problem (2) has at least a nontrivial solution.

**Proof**

From Remark 7 and Steps  1–3, by Weierstrass theorem, the functional *φ* has a critical point, which is just the solution of problem (2).

**Remark**

Let *p* ^−^ > max⁡{*α* ^+^, *β* ^+^, *θ* ^+^} and consider the following two nonsmooth locally Lipschitz functions:

(49) $$\begin{array}{c} F\left(x,t\right)=\{\begin{array}{cc} 0, & 0\leq \left|t\right|<\delta ,\\ \max\left\{{\left|t-\delta \right|}^{\theta (x)},{\left|t-\delta \right|}^{\alpha (x)}\right\}, & t\geq \delta ,\\ \max\left\{{\left|t+\delta \right|}^{\theta (x)},{\left|t+\delta \right|}^{\alpha (x)}\right\}, & t\leq -\delta , \end{array}\\ G\left(x,t\right)=\{\begin{array}{cc} 0, & 0\leq \left|t\right|<\delta ,\\ \max\left\{{\left|t-\delta \right|}^{\theta (x)},{\left|t-\delta \right|}^{\beta (x)}\right\}, & t\geq \delta ,\\ \max\left\{{\left|t+\delta \right|}^{\theta (x)},{\left|t+\delta \right|}^{\beta (x)}\right\}, & t\leq -\delta , \end{array} \end{array}$$

where 0 < *δ* < 1, inf⁡_*x*∈*Ω*_(*α*(*x*) − *θ*(*x*)) > 0, inf⁡_*x*∈∂*Ω*_(*β*(*x*) − *θ*(*x*)) > 0 and *θ* ^−^ > 1. In the following, we will show that *F*(*x*, *t*) satisfies hypotheses *H*(*F*) and (*f*_1_) − (*f*_3_), and *G*(*x*, *t*) satisfies hypotheses *H*(*G*) and (*g*_1_)-(*g*_2_).

Note that *t* ↦ |*t* − *δ*|^*θ*(*x*)^ and *t* ↦ |*t* − *δ*|^*α*(*x*)^ are convex functions; thus, *F*(*x*, *t*) is also convex. Since *t* ↦ |*t* − *δ*|^*θ*(*x*)^, *t* → |*t* − *δ*|^*α*(*x*)^ are locally Lipschitz, so *t* ↦ *F*(*x*, *t*) is locally Lipschitz. Thus, *t* ↦ *F*(*x*, *t*) is regular. Then

(50) ∂F(x,t) ={0,0≤|t|≤δ,θ(x)(t−δ)θ(x)−1,δ<t<1+δ,{λθ(x)+(1−λ)α(x):λ∈[0,1]},t=1+δ,α(x)(t−δ)α(x)−1,t>1+δ,−θ(x)(−t−δ)θ(x)−1,−1−δ<t<−δ,{−λα(x)−(1−λ)θ(x):λ∈[0,1]},t=−1−δ,−α(x)(−t−δ)α(x)−1,t<−1−δ.

Hence, for any *w* ∈ ∂*F*(*x*, *t*), we have

(51) $$\begin{array}{c} \left|w\right|\leq \{\begin{array}{cc} 0, & 0\leq \left|t\right|\leq \delta ,\\ \theta^{+}{\left(t-\delta \right)}^{\theta (x)-1}<\theta^{+} & \\ \;<\theta^{+}{\left(\frac{1}{\delta }\right)}^{\left(\alpha^{+}-1\right)}{\left|t\right|}^{\alpha (x)-1}, & \delta <t<1+\delta ,\\ \theta^{+}{\left(-t-\delta \right)}^{\theta (x)-1}<\theta^{+} & \\ \;<\theta^{+}{\left(\frac{1}{\delta }\right)}^{\left(\alpha^{+}-1\right)}{\left|x\right|}^{\alpha (x)-1}, & -1-\delta <t<-\delta \\ \alpha^{+}+\theta^{+}<\left(\alpha^{+}+\theta^{+}\right){\left|t\right|}^{\alpha (x)-1}, & \left|t\right|=1+\delta ,\\ \alpha^{+}{\left|t\right|}^{\alpha (x)-1}, & \left|t\right|>1+\delta . \end{array} \end{array}$$

Thus, we have

(52) $$\begin{array}{c} \left|w\right|\leq \left(\alpha^{+}+\theta^{+}+{\left(\frac{1}{\delta }\right)}^{\left(\alpha^{+}-1\right)}\right){\left|t\right|}^{\alpha (x)-1},\;\forall w\in \partial F\left(x,t\right),\\ w=\theta \left(x\right){\left(t-\delta \right)}^{\theta (x)-1}\geq \theta^{-}{\left(t-\delta \right)}^{\theta^{+}-1},\;\forall t\in \left(\delta ,1\right]. \end{array}$$

Therefore, conditions *H*(*F*) and (*f*_1_) − (*f*_3_) hold. In a similar fashion, we have that conditions *H*(*G*) and (*g*_1_)-(*g*_2_) hold.
